# Dimensional Fidelity and Orientation Effects of PolyJet Technology in 3D Printing of Negative Features for Microfluidic Applications

**DOI:** 10.3390/mi15030389

**Published:** 2024-03-13

**Authors:** Michael Krause, Analise Marshall, Jeffrey K. Catterlin, Terak Hornik, Emil P. Kartalov

**Affiliations:** 1 MOVES Institute, Naval Postgraduate School, 1 University Circle, Monterey, CA 93943, USA; 2 Physics Department, Naval Postgraduate School, 1 University Circle, Monterey, CA 93943, USAjkcatter@nps.edu (J.K.C.);

**Keywords:** prototyping, photopolymers, precision, accuracy, fidelity, 3D printing, additive manufacturing, embedded, sacrificial material

## Abstract

Negative features in microdevices find a wide range of applications. The process of 3D printing has revolutionized their fabrication due to the combination of good resolution and integration capability. Herein, we report on a systematic study of the effects of materials and print directions on the 3D printing of microfluidic channels as negative features under PolyJet technology. Specifically, the Statasys Objet500 printer was used for this study. We printed two sets of chips (n=10 each), each of which contains channel pairs of a high-contrast reference material and a sacrificial material, respectively. Both materials were embedded in a clear photopolymer resin. The channel pairs ranged in planned width from 64 to 992 μm. To explore the effect on print orientation, channels were printed either parallel or perpendicular with respect to the jetting head’s movement. The width of each channel of a pair was compared for each planned width and each combination of materials. The effect of print orientation on channel morphology was also investigated. We found that reproducibility and accuracy were highest at a planned channel width of approximately ≥600 μm and that channel morphology was most suitable when the jetting head of the printer moved parallel to the channel’s longitudinal axis. The results should be of interest to any users who wish to create negative features using PolyJet 3D technology.

## 1. Introduction

In recent years, 3D printing has revolutionized manufacturing not only for commercial uses but also in fields such as research and development, as well as prototyping in engineering [1,2,3]. One reason that makes 3D printing attractive is the possibility to create highly specialized custom devices. Another reason is the ability to integrate and upscale complex architectures organically as part of the same print. This ability is particularly important to fields such as microfluidics [4,5,6,7], where the production of 3D integrated devices would be too expensive by traditional means [8]. Microfluidic devices contain cavernous microstructures that cover a wide range of applications in biotechnology and medical diagnostics [5,6,9,10,11,12,13,14,15], including artificial muscles [16,17] and biofuel cells [18,19,20]. As a result, the advent of 3D-printed microfluidics is highly impactful to a wide range of fields and applications. The trade-off is that since microfluidic channels are created as negative features in 3D-printed devices, it is necessary to solve the related problems, such as fidelity, consistency, and the removal of sacrificial material. We recently reported on a method [21] to solve the removal problem, specifically within the Stratasys PolyJet technology. Among 3D printing techniques, PolyJet is particularly significant as it combines high resolution, large print volume, ability to print soft resins, and the ability to print multiple resins at the same time in the same device. So, its characterization is of high practical importance.

Here, we present a systematic investigation that is of practical significance to users of 3D printing, as it studies the behavior and performance of a Stratasys PolyJet printer under different conditions. Specifically, this study compares the physical dimensions of microfluidic channels set in the STL file (i.e., the planned width of a channel) to those measured in the printed materials. Previous work indicated that PolyJet-printed material is not within the accuracy limits as claimed by the manufacturer [22]. Moreover, our previous work on the PolyJet sacrificial material clearance procedure established that the technology prints sacrificial material and resin features generally wider than planned [21]. This exposed a need for proper calibration, to allow for high-quality predictive engineering of such structures, while also exploring additional fabrication factors, such as design orientation in reference to the printing head axes.

The effects of print direction, layer thickness, and material on accuracy and predictability, using PolyJet or other technologies, had previously been assessed [8,11,22,23,24,25,26,27,28,29] and reviewed [30]; however, neither the level of detail nor the focus of these studies is sufficient to address our specific problem of microfluidic channel printing. For instance, several studies on accuracy do not consider microfluidic channels [22,25,26,27,28]. Further, while Rebong et al. and co-workers [27] compare the print accuracy of different print technology platforms, they do not compare the effect of jetting head print direction within a single technology. Similarly, many 3D printing accuracy studies (e.g., [23,24]) systematically compare multiple print parameters, but do not focus on its effects on negative features or microchannel printing specifically. Macdonald et al. [11] compare in detail how different print technologies, including PolyJet, affect laminar flow in microchannels. Solutions in applications of our chips (biofuel cells, artificial muscles) are stationary and neither flow nor need to mix. Lastly, the present study explores the printing effects of different materials, which is an aspect many other studies did not consider [27,28].

## 2. Materials and Methods

***Chip Fabrication***. All chips were printed on our 3D printer Stratasys Objet500 Connex 1 (Stratasys, Rehovot, Israel). Chips were initially designed in the 3D CAD design software SOLIDWORKS 2022 (SolidWorksCorp., Waltham, MA, USA) and then converted for compatibility with the Objet500 Connex 1. The digital models were laid flat on the print bed and were oriented so that for one set of chips, the printer’s jetting head ran parallel, and for the other, the head ran perpendicular to the longitudinal axis of the microfluidic channels. Both sets of chips were printed in batches.

**Figure 1:**
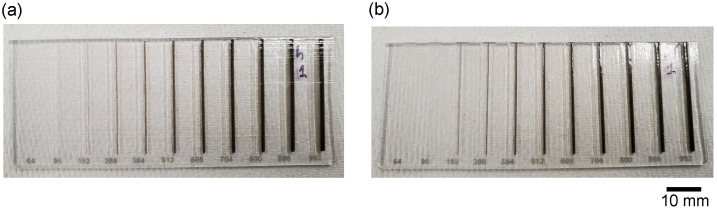
Samples of chips printed in two different orientations. Each chip contains 11 pairs of channels grouped by matching planned width. For each pair, the channel on the left contains sacrificial material and the one on the right contains black reference material. The width of each channel in a pair is printed at the bottom of each chip (in μm). (**a**) Sample of a chip where orientation of the longitudinal axis of the channels was parallel with respect to the jetting head’s movement. (**b**) Sample of a chip where the orientation of the longitudinal axis of the channels was perpendicular with respect to the jetting head’s movement. The upper right corner of each chip reflects the surrounding light. That reflection reveals the texture on the chips’ surface, which is a result of the print orientation. The scale bar applies to (**a**,**b**).

***Chip Architecture***. Chips were designed as a rectangular prism with dimensions 90 mm × 35 mm × 1.75 mm (length, height, depth). Each chip contained 11 pairs of internal channels. The width of each channel was a multiple of 32 μm (minimum layer thickness of the printer’s *z*-axis in multi-material mode is 30 μm [31]). Empirically the layer thickness of the Object 500 Connex in multi-material mode was found to be 43 μm. All chips are printed in multi-material mode and the layer thickness was constant throughout this study. The planned individual widths were 64, 96, 192, 288, 384, 512, 608, 704, 800, 896, and 992 μm (Figure 1). Channels were either printed with the longitudinal axis parallel to the jetting head’s *x*-axis (parallel printing, Figure 1a) or with the channel’s longitudinal axis perpendicular to the jetting head’s *x*-axis (perpendicular printing, Figure 1b).

***Materials***. Three different photopolymer materials (Stratasys, Eden Prairie, MN, USA) were used in this study. The clear resin chip was printed using VeroClear-RGD810. The material was chosen based on its optical clarity, chemical resistance, and rigidity. SUP706B was used as the sacrificial material. Agilus30 Black FLX985, a black resin, was used for the reference channels, because it provides a good optical contrast. All chips were printed monolithically in a single process, with SUP706B and Agilus30 Black FLX985, respectively, embedded in VeroClear-RGD810 to form the channel pairs.

***Baking the Chips***. Previous work [21] has shown that exposing the sacrificial material to 80 °C leads to the formation of microcavities. A clearance procedure [21] exploits that effect to allow the clearance solution to fill those microcavities and helps to dislodge the sacrificial material. Thus, baking chips at 80 °C for approximately 2–4 h is part of the standard clearance procedure [21]. All measurements in this study were taken before and after baking the chips.

***Mass loss measurements***. The oven was preheated to 45 °C and 80 °C, respectively. For each temperature condition, n=20 Eppendorf tubes were labeled, filled with 0.5 ± 0.2 g of SUP706B resin, and weighed prior to heating. The tubes were fixed upright in a perforated holder with lids open and placed in the preheated oven for two hours at the respective temperatures. The tubes were removed from the oven after two hours and allowed to cool to room temperature. The tubes were then sealed by closing their lids and weighed to determine their final mass.

***Optical setup and imaging***. Optical imaging was conducted on a binocular stereo zoom microscope system with a 0.6–3.2× magnification (SM-1TZ-PL-10MA, AmScope, United Scope LLC, Irvine, CA, USA). Images were taken with an attached 10 MP color camera. Mineral oil (J217-500ML, VWR Life Science) was routinely added to the chips’ surface to smooth out surface roughness from the printing process and reduce variations in the refractory index. Channels were inspected and an image was taken for each channel type of a pair (sacrificial material or reference channel), for each width, and for each chip at 1.6× magnification. We calibrated the imaging software by taking an image of a 1 mm stage micrometer slide with 10 µm unit spacing that was imaged at the same magnification as the specimen. The scaling tool of the AmScope software was used to measure the width of each channel of a pair. Channels of 64 μm planned width were excluded from the analysis, because the channel could not be discerned from the image background. Five chips were cut along their long axis to expose the channel cross-sections. Images of channels in those cut chips were taken with an inverted microsope (Olympus IX50, Tokyo, Japan), taken at 4× magnification, and analyzed using ImageJ software [32].

***Analysis of printing accuracy and reproducibility***. Width data obtained as described in the previous paragraph were aggregated in spreadsheets. The relative error of the width measurement, ${\mathrm{Err}}_{rel}$, was defined as

$${\mathrm{Err}}_{rel}=\frac{W_{m}-W_{s}}{W_{s}},$$

where $W_{s}$ denotes the planned width and $W_{m}$ denotes the measured channel width, respectively. All data were tabulated in spreadsheets and custom Python scripts were used to analyze, summarize, and visualize the data. Data points for a given planned width represent the mean (n=10) and its standard deviation, represented as an error bar around the mean.

## 3. Results and Disussion

### 3.1. Mass Loss of SUP706B during Baking

**Figure 2:**
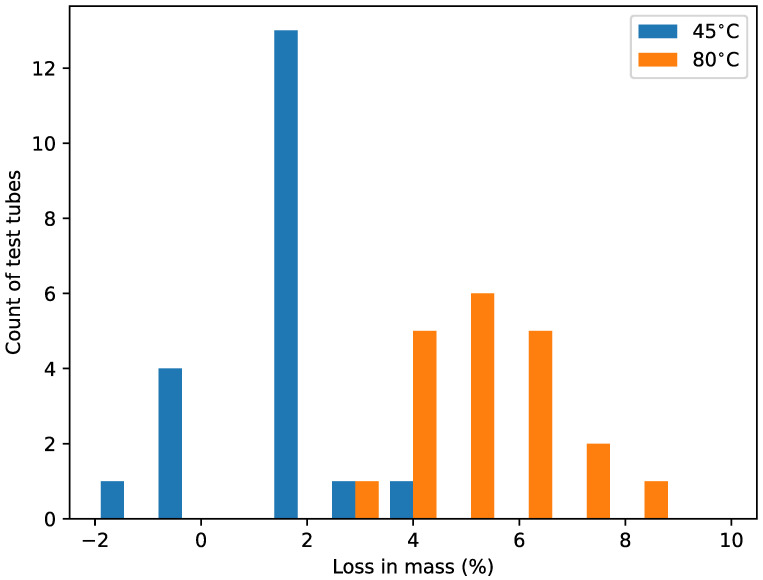
Histogram showing the loss in sacrificial material (SUP706B) mass following baking at 45 °C (blue bars, 1.28±1.16%, mean ± S.E.M.) and 80 °C (orange bars, 5.35±1.18%, mean ± S.E.M.), respectively. Bin width 0.2%.

To systematically test the effect of baking on the core chip material, an experiment was conducted where samples of SUP706B (n=20 in each group) were exposed to two different temperatures and the mass of each sample was measured before and after baking [21]. Melting, i.e., the transition from solid to a liquid, was not observed during heating. A loss in mass was observed after baking at 45 °C of 1.28±1.16% and a loss in mass after baking at 80 °C of 5.35±1.18% (mean ± standard deviation of the sample). An increase in mass was noted at 45 °C. That increase might be explained by the material having absorbed water vapor from the atmosphere after baking and cooling. It may have happened at 80 °C as well, but the loss is so much larger that it drowns mass increase from the absorption. Using a Student’s *t* test, we rejected the null hypothesis that the samples taken at the two temperatures are from the same population (t=−11.03, $p\leq 10^{-10}$). We conclude that the change in mass recorded and observed appeared to be through a degassing process (Figure 2).

### 3.2. Examples of the Measurement Procedure

**Figure 3:**
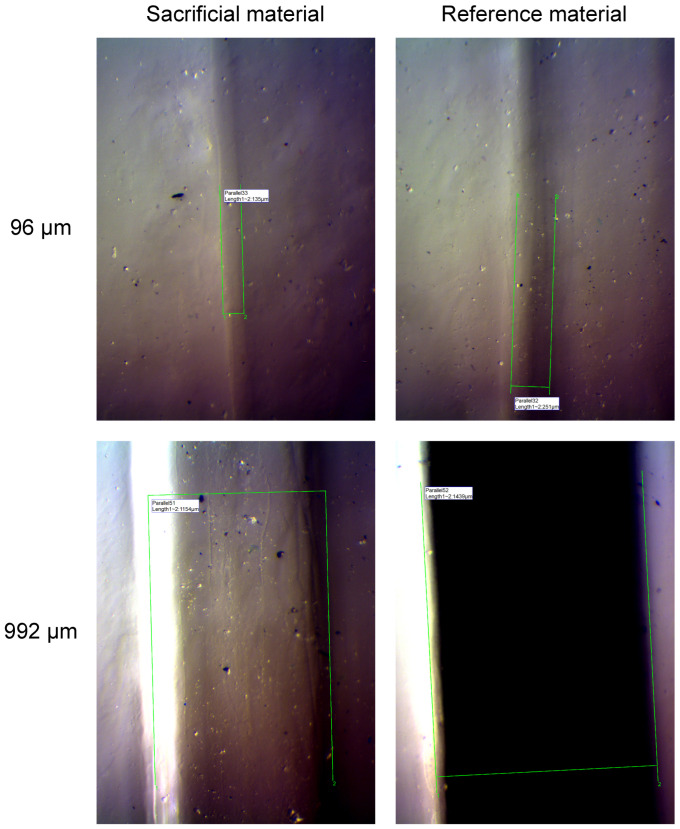
Examples of the channel width measurement procedure on a chip where the printer’s jetting head was oriented parallel with respect to the longitudinal axis of the channels. Images shown contain either sacrificial material or black reference material (refer to labels at the top), after baking. The shortest (96 μm) and longest (992 μm) planned channel width analyzed is indicated on the left. The green lines in each image and the white box that contains the measured distance in μm are added to the image by the imaging software’s measurement tool. All images are at the same scale.

Ten (n=10) chips were printed for each orientation, and each chip contained m=11 channel pairs. First, the chips were analyzed by inspecting the channels of each pair under the microscope and then imaging software was used to measure the width of each channel. Figure 3 shows examples of the measurement procedure for the shortest and the longest planned width. The image analysis system allowed easy positioning of measurement points on the images. Note that the width of each of the four channels measured was consistently larger than their planned widths.

### 3.3. Print Orientation Affects the Dimensional Precision and Channel Morphology

**Figure 4:**
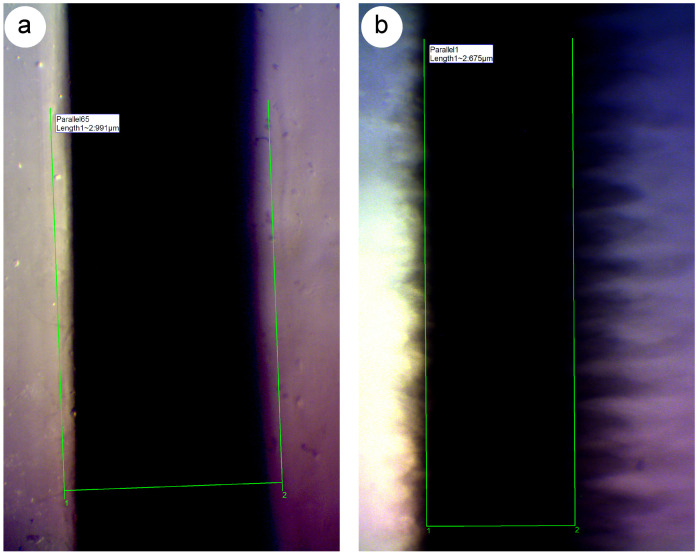
Direct comparison of channels printed in different orientations. (**a**) Channel printed where the printer’s jetting head was oriented parallel with respect to the longitudinal axis of the channels. (**b**) Channel printed where the printer’s jetting head was oriented perpendicular with respect to the longitudinal axis of the channels. Planned width of channels in (**a**,**b**) was 608 μm and both channels have been embedded with black reference material. Images in (**a**,**b**) are at the same scale.

To compare the effect of the relative movement of the printer’s jetting head on the channel’s dimensional precision, accuracy, and morphology, we printed one set of chips where the direction of movement of the printer’s jetting ran parallel to the channel’s longitudinal axis (Figure 4a, parallel orientation), and another set of chips where the direction of the jetting head ran perpendicular to the channel’s longitudinal axis (Figure 4b, perpendicular orientation). Figure 4 shows how the print direction affects the measured width and the overall channel morphology.

Figure 4 only shows a channel embedded with black reference material. However, a similar difference in print orientation was observed in channels embedded with sacrificial material. The effect was more clearly visible when channels were embedded with black reference material. Note the smooth interface between the black reference material and the clear resin chip material when the printer’s jetting head moved parallel to the channel’s longitudinal axis (Figure 4a). In that situation, the printer would deposit material in one sweep along the entire length of the channel.

By contrast, when the printer’s jetting head moved perpendicular to the channel’s longitudinal axis (Figure 4b), the interface between the black reference material and the core chip material appeared jagged, suggesting a rough surface of the inner lining of the channel. The same observation of a jagged inner channel lining was made in channels with sacrificial material, but Figure 4 shows only the black reference material for its higher contrast. Chips printed with sacrificial material show a similar jagged appearance when printed in the perpendicular orientation. This can be seen, for all images of channel width ≥ 608 μm, particularly in chips with sacrificial material.

In that print orientation, the printer needs to swap core with embedding material at an exact distance in order to deposit black reference material. It then deposits black reference material for a very short distance (i.e., the planned width of the channel). Thus, it is plausible that this process is mechanically challenging, e.g., due to backlash error, and thus produces the jagged interface between the two materials. These data show that the print orientation affects the physical structure and channel morphology, and consequently print reproducibility.

Dependence of surface roughness on print orientation has been reported [26,29,30,33], and Thakare et al. summarized how print orientation affects the finished part using PolyJet technology. The results suggest that the surface roughness of the inner lining of the microchannel, made in all cases from VeroClear-RGD810, is higher when the printer’s jetting head is oriented perpendicular to the longitudinal axis of the channel. For the purpose of manufacturing parts that need to be cleared from sacrificial materials, the preferred orientation is when the jetting moves parallel to the microchannel. The effect of print orientation on surface roughness could be of interest to users of applications in which laminar flow is important. But the target applications for our research are artificial muscle construction and bacterial biofuel cells. In those applications, surface roughness is not an important factor as the fluid in the microchannel does not flow or mix during operation, but is stationary. Furthermore, the chips created in this study have microchannels embedded and the investigation of the channel topology and the process of cutting chips open is involved. For these reasons, we decided not to perform a more detailed analysis of this observation at this point (e.g., scanning electron microscopy).

### 3.4. Comparing Planned Channel Width to Measured Channel Width

**Figure 5:**
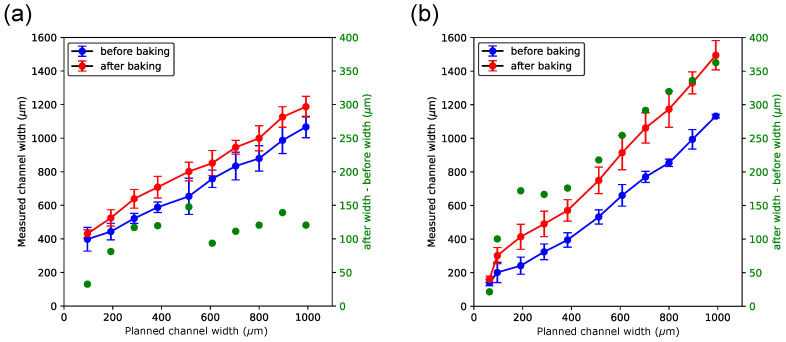
Measured widths of microfluidic channels when channels are printed parallel to the printer’s *x*-axis. Channels contain either sacrificial (**a**) or black (**b**) reference material. In both panels, measurements of channel widths shown in blue color were taken before and those shown in red color were taken after chips had been baked, respectively. Shown in green color (right *y*-axis in each panel) is the difference in channel width between after baking and before baking.

**Figure 6:**
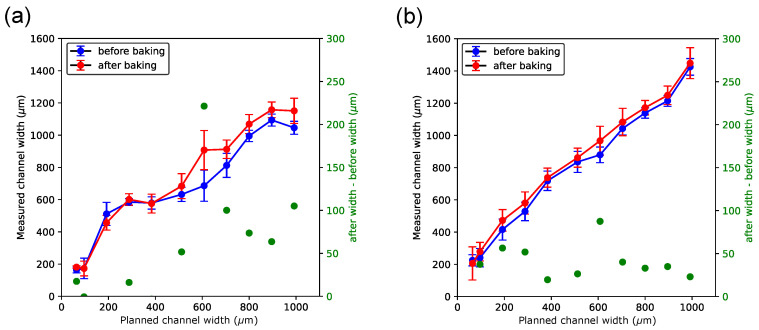
Measured widths of microfluidic channels when channels are printed perpendicular with respect to the printer’s *x*-axis. Channels contained either sacrificial (**a**) or black (**b**) reference material. In both panels, measurements of channel widths shown in blue color were taken before and measurements shown in red color were taken after chips had been baked, respectively. Shown in green color (right *y*-axis in each panel) is the difference in channel width after baking and before baking.

Figure 5 and Figure 6 show the measured width plotted vs. the planned width. The measurement values before baking of the chips are shown in blue, while those after baking are shown in red. The mass loss of sacrificial material during baking suggested outgassing, which could produce a ballooning effect that would explain the observed widening of the features following baking (see Section 2, Materials and Methods and Figure 2).

This effect can consistently be seen in chips where the printer’s jetting head moved parallel to the longitudinal axes of the channels (Figure 5), but appears more pronounced in channels embedded with the black reference material (Figure 5b) than in those embedded with sacrificial material (Figure 5a). The difference in width (shown in green ink) shows that the relative widening of the features decreases with increasing width for the sacrificial material (Figure 5a), while it increases at a constant rate with increasing width for the black reference material (Figure 5b).

A possible explanation for that observation is that the Agilus30 Black FLX985 material is even more prone to developing microcavities following baking compared to the sacrificial material. The effect of baking on the measured channel width is expressed in green color in both figures and shows that the expansion effect is more constant (i.e., less dependent on the planned width) in channels embedded with sacrificial material.

Measurement of the channel width in chips where channels were printed perpendicular to the longitudinal axes of the channels (Figure 6) was more challenging compared to the parallel print orientation. In these chips, the interface between either sacrificial or reference material and the clear resin appeared jagged, akin to a zigzag pattern (see Figure 4), as described in the Results Section 3.3. This effect was particularly pronounced in channels where sacrificial material was embedded (Figure 6a), and it made the measurement more challenging.

Figure 6a shows that the relationship of the measured width versus the planned width is not as linear for microchannels of the same materials but printed in a different orientation (Figure 5a). Moreover, the relatively large variations in the measurement errors seen in Figure 6a are presumably a consequence of the challenging width measurement. In that print orientation, the difference between the channel width before and after baking per data point was relatively small compared to that seen in Figure 5 (right ordinate, in green ink).

From the literature, it is not clear what print orientation in 3D printing is generally preferred to achieve maximum accuracy [26,29,30,33]. The present results suggest that for the applications of interest in this study (microfluidic channels, artificial muscle, biofuel cells), moving the printer’s jetting parallel to the longitudinal axis of the channel is preferred.

### 3.5. The Magnitude of the Error of the Measured Width Depends on the Planned Width

**Figure 7:**
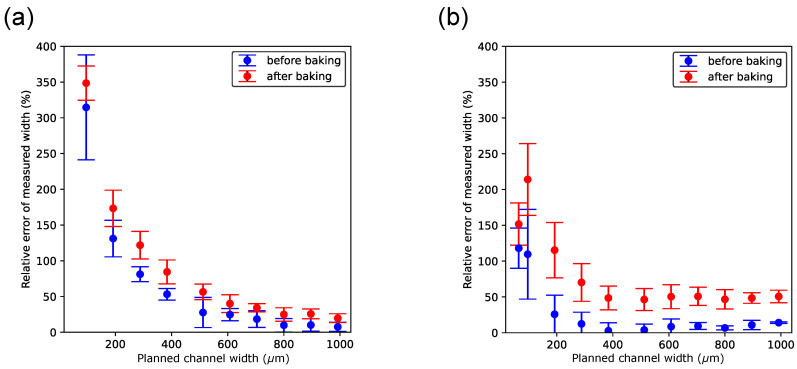
Relative error of the width measurement as a function of the planned channel width when channels are printed in the parallel orientation. (**a**) Channels filled with sacrificial material. (**b**) Channels embedded with black reference material. In both panels, measurements of channel widths shown in blue color were taken before and measurements shown in red color were taken after chips had been baked, respectively.

**Figure 8:**
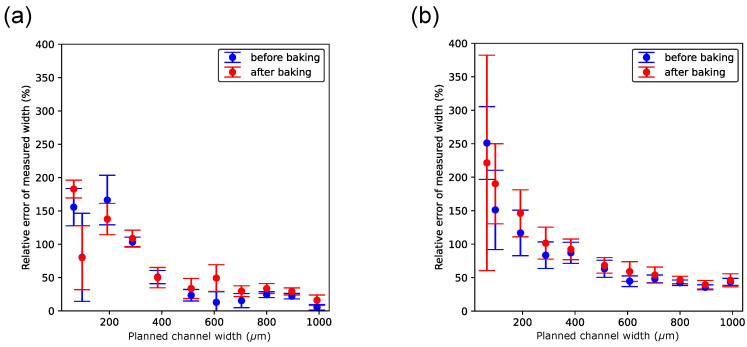
Relative error of the width measurement as a function of the planned channel width when channels are printed in the perpendicular orientation. (**a**) Channels embedded with sacrificial material. (**b**) Channels embedded with black reference material. In both panels, measurements of channel widths shown in blue color were taken before and measurements shown in red color were taken after chips had been baked, respectively.

The foremost motivation for conducting this study was to learn more about the dimensional accuracy of the Objet500 PolyJet printer. The main hypothesis was that the accuracy of the print dimensions decreases as the planned distances in 3D objects decrease, e.g., the dimensions of negative features. To test this hypothesis, an analysis of the relative error of the width measurement was performed (see Section 2, Materials and Methods, for the definition). Figure 7 and Figure 8 show that the relative error generally decreases with increasing planned width. The rate of decrease in that error becomes relatively small starting at a planned width of approximately 600 μm, regardless of the print orientation or whether the channel width had been measured before or after baking. This width and error magnitude is reasonable and within the range of the minimum microchannel size found in other studies using PolyJet technology (e.g., [11,26]), given that the effective layer thickness in this study is 43 μm.

When the printer’s jetting head was oriented perpendicular to the channel’s longitudinal axis (Figure 8), the data points for the error showed higher standard deviations, particularly at the smaller planned width. This was expected given the challenging measurement conditions with that print orientation. The error between the planned and measured widths is overall smaller when the printer’s jetting head moves parallel to the longitudinal axis to the channel. This result provides further reason to give this print direction preference.

## 4. Analysis of Channel Widths and Heights from Cross-Sections

**Figure 9:**
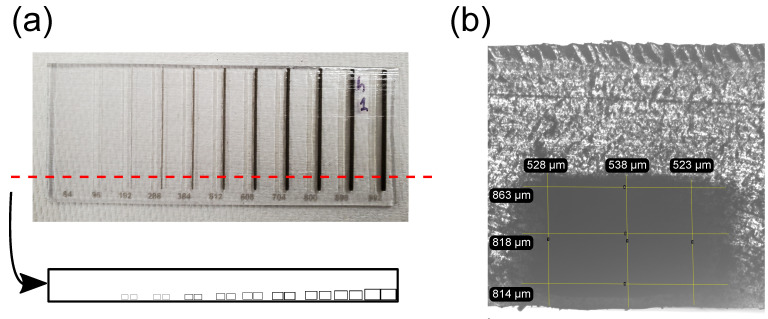
Creation of chip cross-sections and measurements of channel width and height. (**a**) Chips were cut along the dashed red line. Subsequently, the cut edge (lower part of the figure) was placed on the microscope stage and each channel’s cross-section was imaged. (**b**) Example image of a channel cross-section with three height and three width measurements.

**Figure 10:**
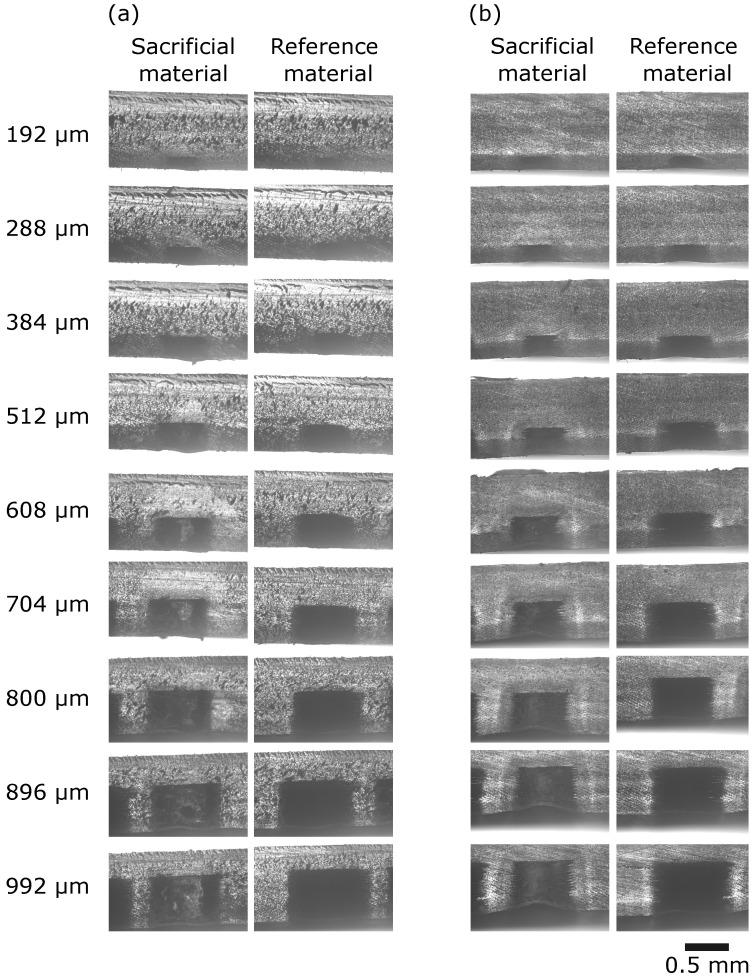
Example images of chips cross-sectional edges from both print orientations. The planned width is indicated on the left for both print directions. The scale bar applies to all images. (**a**) Parallel print orientation. (**b**) Perpendicular print orientation.

Next, we investigated channel dimensions in cross-cut sections (n=5 for each print direction). Figure 9 shows how cross-cuts were produced and how the channel height and width were measured. Figure 10 shows example images of each print direction and each planned width. Although each chip has 11 channels, we only analyzed and show 9 because the channels of planned widths 64 μm and 92 μm in the cross-section cuts were not discernable and were excluded from the analysis for that reason.

**Figure 11:**
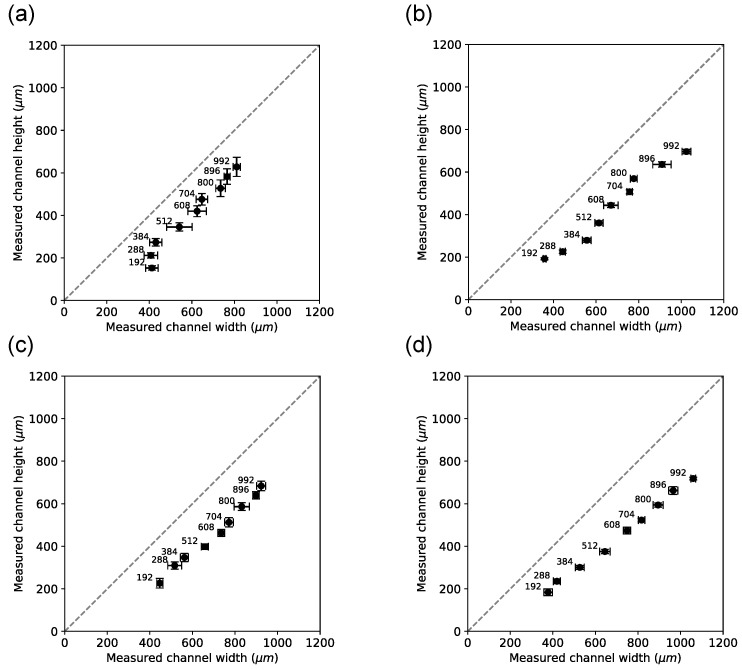
Analysis of cross-section measurements of channel width and height. Planned channel width (in μm) is indicated to the upper left for each data point. Each data point represents an average of n=5 chips for a given planned channel width. Error bars represent the standard deviation of the sample. The dashed line of the unity slope is given as reference in each panel. (**a**) Channels printed with sacrificial material in the perpendicular orientation. (**b**) Channels printed with black reference material in the perpendicular orientation. (**c**) Channels printed with sacrificial material in the parallel orientation. (**d**) Channels printed with black reference material in the parallel orientation.

**Figure 12:**
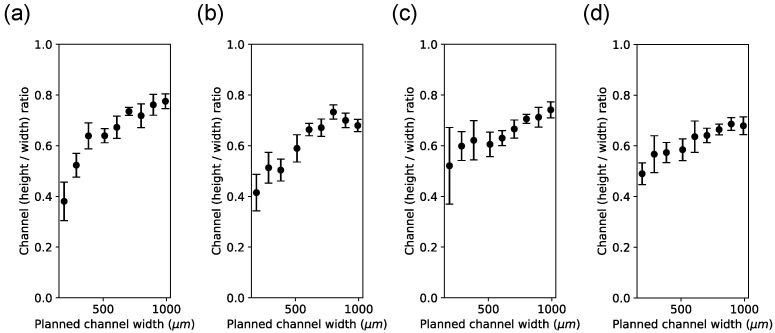
Channel side/length ratios from cross-sections. All graphs show the ratio of height over width plotted against the planned channel width. Each dot represents the average ratio (n=5) plotted against the planned channel width. The widths range from 192 μm to 992 μm and are the same as shown in Figure 11. Error bars represent the standard deviation of the sample. (**a**) Channels printed with sacrificial material in the perpendicular orientation. (**b**) Channels printed with black reference material in the perpendicular orientation. (**c**) Channels printed with sacrificial material in the parallel orientation. (**d**) Channels printed with black reference material in the parallel orientation.

Figure 11 shows the measured height plotted against the measured width for each channel, each material, and each print direction. Channel height was consistently lower compared to channel width. This result was confirmed when the height/width ratio was computed (Figure 12). A possible explanation for the lower channel height compared to the channel width is that channels were printed close (approx. 60 μm) from the chip’s surface. That proximity to the surface permits good microscopic inspection of the channel width in the top view. However, printing close to the chip’s surface may have printed the channels with lower height than planned. The planned heights in this figure are indicated by the label next to the data point in Figure 11. The planned heights’ data points correspond better to their measured heights at lower planned height values, but at planned higher values, the measured heights are consistently smaller than the planned heights, starting at approximately 500 μm.

Figure 12 shows that the ratio approaches unity as the planned width increases, suggesting that print accuracy becomes progressively less predictable with decreasing planned height.

**Figure 13:**
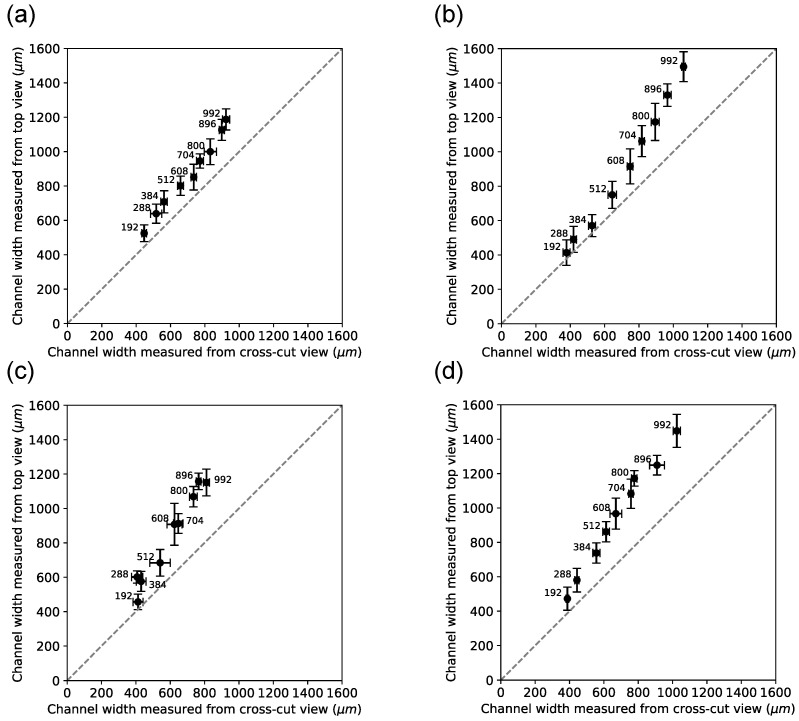
Analysis of cross-section channel width measurements (on the abscissa) compared to top-view channel width measurements (on the ordinate). Planned channel width (in μm) is indicated to the upper left for each data point. Each data point represents an average of n=5 (cross-section view) and n=10 (top view) chips for a given planned channel width. Error bars represent the standard deviation of the sample. The dashed line of the unity slope is given as reference in each panel. (**a**) Channels printed with sacrificial material in the perpendicular orientation. (**b**) Channels printed with black reference material in the perpendicular orientation. (**c**) Channels printed with sacrificial material in the parallel orientation. (**d**) Channels printed with black reference material in the parallel orientation.

Figure 13 demonstrates the correspondence of channel widths for each material–orientation combination, measured by the two approaches taken in this study (top-view and cross-sectional view, respectively). The top-view widths data are those shown in Figure 5 and Figure 6, and only data are shown from chips after baking. Planned channel widths (indicated by the label on each data point) correspond better with widths values as the planned widths increase.

## 5. Conclusions

Herein, we report on the experimental characterization of dimensional fidelity, consistency, and orientation effects of PolyJet technology in the hybrid 3D printing and fabrication of negative features using our already reported method of sacrificial material removal. A practical value of the presented results is that it gives PolyJet users guidance when engineering devices. It recommends the planned dimensions to be entered in order to ensure that the output satisfies the desired physical dimensions. Furthermore, the relative error results can guide users to make educated trade-offs in the engineering of their designs. The reported results are important to 3D-printed microfluidics in particular, and the 3D printing of negative features in general.

## Data Availability

Data are contained within the article.

## Abbreviations

STL	Standard Tessellation Language
CAD	Computer-aided Design
